# Predicting Bison Migration out of Yellowstone National Park Using Bayesian Models

**DOI:** 10.1371/journal.pone.0016848

**Published:** 2011-02-14

**Authors:** Chris Geremia, P. J. White, Rick L. Wallen, Fred G. R. Watson, John J. Treanor, John Borkowski, Christopher S. Potter, Robert L. Crabtree

**Affiliations:** 1 Yellowstone Center for Resources, National Park Service, Yellowstone National Park, Wyoming, United States of America; 2 Natural Resource and Ecology Laboratory, Colorado State University, Fort Collins, Colorado, United States of America; 3 Watershed Institute, California State University Monterey Bay, Seaside, California, United States of America; 4 Department of Mathematical Sciences, Montana State University, Bozeman, Montana, United States of America; 5 Ames Research Center, Moffett Field, California, United States of America; 6 Yellowstone Ecological Research Centre, Bozeman, Montana, United States of America; University of California, Berkeley, United States of America

## Abstract

Long distance migrations by ungulate species often surpass the boundaries of preservation areas where conflicts with various publics lead to management actions that can threaten populations. We chose the partially migratory bison (*Bison bison*) population in Yellowstone National Park as an example of integrating science into management policies to better conserve migratory ungulates. Approximately 60% of these bison have been exposed to bovine brucellosis and thousands of migrants exiting the park boundary have been culled during the past two decades to reduce the risk of disease transmission to cattle. Data were assimilated using models representing competing hypotheses of bison migration during 1990–2009 in a hierarchal Bayesian framework. Migration differed at the scale of herds, but a single unifying logistic model was useful for predicting migrations by both herds. Migration beyond the northern park boundary was affected by herd size, accumulated snow water equivalent, and aboveground dried biomass. Migration beyond the western park boundary was less influenced by these predictors and process model performance suggested an important control on recent migrations was excluded. Simulations of migrations over the next decade suggest that allowing increased numbers of bison beyond park boundaries during severe climate conditions may be the only means of avoiding episodic, large-scale reductions to the Yellowstone bison population in the foreseeable future. This research is an example of how long distance migration dynamics can be incorporated into improved management policies.

## Introduction

The approximately 3,900 bison in Yellowstone National Park (Yellowstone) represent the largest free-ranging population of plains bison in North America; a remnant of the millions of bison that once roamed the continent [1]. After near extirpation in the early twentieth century, Yellowstone bison were restored from fewer than 25 individuals through intense husbandry and within park reintroductions through 1938, after which abundance was limited by regular culling [2]. The park ceased culling in 1969 and allowed numbers to fluctuate in response to weather, predators, and resource limitations. The population grew to about 5,000 animals in 2005 and, as numbers increased, seasonal migrations along altitudinal gradients began, with bison moving from higher-elevation summer ranges to lower-elevations during winter, and returning to summer ranges during June and July.

Range expansion may delay responses to food limitation such as diminished survival and fecundity until new areas can no longer be colonized to provide additional forage [3]. Expansion of the winter range areas used by Yellowstone bison was detected in the 1980s and contributed to sustained population growth. More bison began migrating earlier and migration distances expanded as density increased [4], [5]. This expansion was amplified when winter weather was severe, likely owing to reduced food availability and increased energetic costs [6], [7]. Yellowstone bison eventually began using winter ranges outside the park, with >98 animals entering the state of Montana each winter after 1988. However, range expansion much beyond the park boundary was precluded by intense management intervention due to concerns of brucellosis transmission to cattle in the greater Yellowstone system. Approximately 60% of the bison population has been exposed to brucellosis, a bacterial disease caused by *Brucella abortus* that may induce abortions or the birth of non-viable calves in livestock and wildlife [8]. When livestock are infected it also results in economic loss from slaughtering infected cattle herds and imposed trade restrictions. Therefore, all bison leaving Yellowstone were hazed (i.e., moved) back into the park by riders on horseback, all-terrain vehicles, or helicopters; harvested by hunters; captured and transported to slaughter; or captured and confined in fenced paddocks until release in spring [9], [10].

The United States government and the state of Montana agreed to an Interagency Bison Management Plan in 2000 that established guidelines for cooperatively managing the risk of brucellosis transmission from Yellowstone bison to cattle and conserving bison as a natural component of the ecosystem, including allowing some bison to migrate out of the park [9]. However, numbers of bison exiting the park far exceeded expectations and approximately 3,700 animals were culled during 2001–2009. Culls were non-random [11], [12], which could have adverse demographic and genetic effects if continued over the long term [1], [13]. The successful, long-term conservation of Yellowstone bison depends on migration to lower-elevation winter ranges in and adjacent to the park [14]. Thus, there was a need to improve predictions of the magnitude of migrations and provide managers with a tool for making informed decisions regarding tolerance for bison in cattle-free areas outside the park and numbers of bison that should be managed in the park.

There have been several efforts to predict the movements of bison outside park boundaries using aerial count data and coarse-scale climatic indicators [6], [7], [15]. Counts are subject to measurement error and underlying processes may be inaccurately evaluated [16]. The hierarchal Bayesian framework provides a coherent structure for assessing uncertainty that arises from errors in observations and variance in the processes being modeled. Bayesian methods treat states or the unobserved true response of interest as random variables [17]. Therefore, these techniques allow us to provide park managers with explicit probabilistic statements of future states, which in this case relates to articulating the probability that the total number of bison outside the park will be within a specified range.

A linear relationship between peak numbers of bison exiting the park, population size, and snow pack development has been suggested [6], [7]. However, numbers migrating cannot exceed population size indicating relationships with density and climatic indicators must be nonlinear. Also, Yellowstone bison function as two semi-distinct breeding herds [2], [18], [19] and out-of-park migrations likely occur at this scale. The central and northern herds exhibit differential movement to the northern and western park boundaries and are exposed to different snow pack and vegetation phenology regimes. We developed mechanistic nonlinear models of migration and used our top supported models to illustrate how long distance migration dynamics could be used to inform policy makers of potential migration scenarios for varying levels of population abundance.

## Materials and Methods

### Study Area

**Figure 1:**
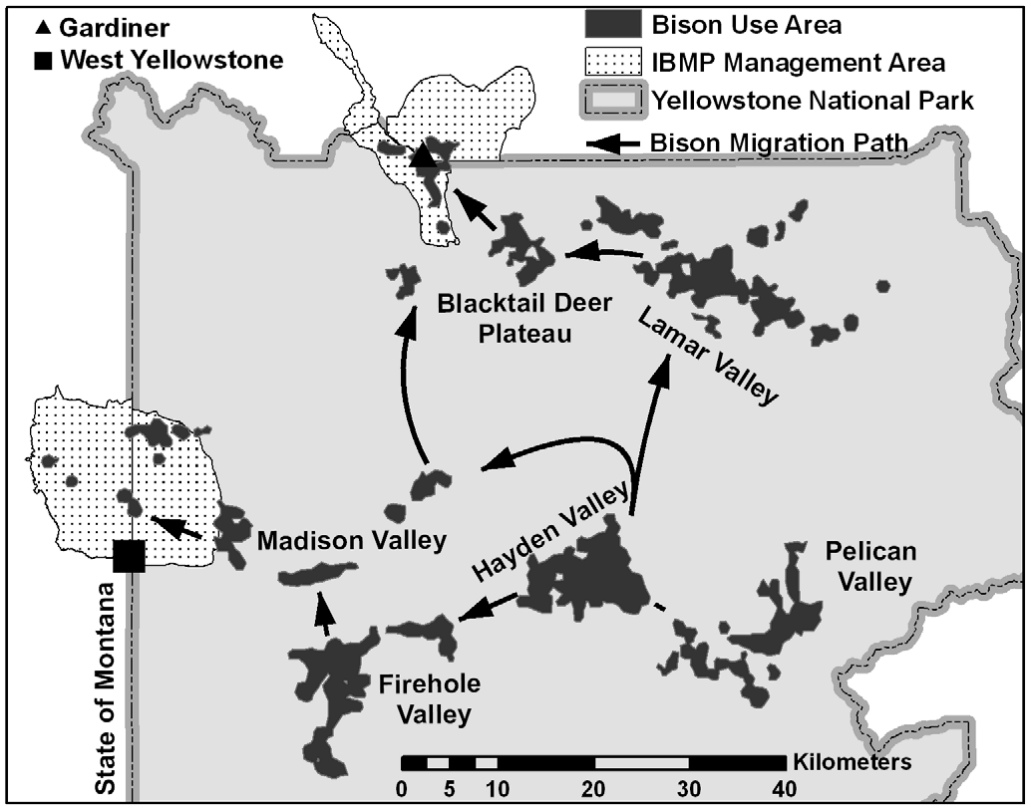
Major use areas of bison in Yellowstone National Park including bison management zones identified in the Interagency Bison Management Plan beyond which bison were rarely observed during 1990–2009.

The central bison herd occupies the central plateau of Yellowstone, which extends from the Pelican and Hayden valleys with a maximum elevation of 2,400 min the east to the lower-elevation and thermally-influenced Madison headwaters area in the west (Figure 1). Winters are severe, with snow water equivalents (i.e., mean water content of a column of snow) averaging 35 cm and temperatures reaching -42 C. The northern herd occupies the comparatively drier and warmer northern portion of Yellowstone. Elevation decreases from 2,200–1,600 m over approximately 90 km between Cooke City and Gardiner, Montana with mean snow water equivalents decreasing from 30 to 2 cm along the east-west elevation gradient.

Bison from the central herd congregate in the Hayden Valley for the breeding season (15 July–15 August), but move between the Madison, Firehole, Hayden, and Pelican valleys during the rest of the year. Also, some bison from the central herd travel to the northern portion of Yellowstone during winter and commingle with the northern herd, with most returning to the Hayden Valley for the subsequent breeding period. Bison from the northern herd congregate in the Lamar Valley and on adjacent high-elevation meadows to the south for the breeding season, but move west towards lower-elevation areas nearer Mammoth, Wyoming and Gardiner, Montana during winter.

### Observations of Responses and Covariates

We considered 142 aerial counts of bison completed near the northern and western boundaries of Yellowstone during October–May, 1990–2009. We counted all bison that were outside the park boundary or within a 5-km buffer inside the park boundary to account for animals that had left the park, were poised to leave the park, or had possibly been hazed back inside the park prior to counting. We summed these counts with the total number of bison that had migrated beyond the park boundary and were culled prior to counting to improve our measure of migration. Culls included bison that were harvested by hunters, shot by agency personnel, moved to out-of-park research or quarantine facilities, sent to slaughter, or held in fenced paddocks until release during spring. Culls were known for each year and aerial surveys provided accurate estimates of numbers because bison are highly visible and often congregate in large groups in open areas [20]. We defined two responses measuring migration since herds differentially move towards each park boundary and are exposed to different climate conditions. Y_N,t_ where 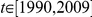 was our observation of migration beyond the northern boundary and was represented as the annual maxima of counts of bison near the northern boundary and culls occurring prior to counting. Y_W,t_ was our observation of migration beyond the western boundary and defined as the annual maxima of counts of bison near the western boundary and culls occurring prior to counting.

Covariates were defined for density, snow pack severity, and aboveground dried biomass. We completed annual breeding season counts of the northern and central herds during July-August, 1990–2009 as a surrogate for density. Bison located in the Madison, Firehole, Hayden, and Pelican valleys were considered part of the central herd, while bison on the Mirror Plateau and in the upper Lamar River valley were included in the northern herd. We defined x_central,t_ as the annual count of central herd animals and x_north,t_ as the annual count of the northern herd. We used a validated snow pack simulation model [21] to estimate daily snow water equivalents (SWE; m) by averaging SWE values across all 28.5×28.5 m pixels within a 99% kernel of bison use [12]. We summed daily model-generated averages during 1 October through 31 April [22], and created single accumulated annual values for the northern range (x_snowN,t_), central interior (x_snowC,t_), and entire park (x_snow,t_). We generated aboveground dried biomass (g/m^2^) estimates using modeled monthly net primary productivity from NASA's Carnegie-Stanford-Ames-Approach (CASA) [23], [24]. CASA, a biophysical ecosystem model, incorporates temperature, precipitation, solar radiation, vegetation cover, and the normalized differential vegetation index (NDVI) from Landsat satellite data as inputs during the April to October growing season [25], [26]. We considered all pixels within the 99% kernel of bison use, except for forested areas that were clipped from analysis because bison are predominantly grazers. The resulting analysis area consisted of approximately 33 meadows greater than 1 km^2^ in size and distributed across the elevation gradient of the northern and central ranges. We censored areas affected by cloud cover within years, resulting in marginal differences in the size of the analysis area between years. Due to this difference, we summed values across available pixels for each year and divided by the number of pixels. We defined x_forageN,t_ for the northern range, x_forageC,t_ for the central interior, and x_forage,t_ for the entire park. The covariate does not exactly reflect annual plant biomass production over the growing season or standing biomass available for wintering bison due to herbivore off take during April through October. However, our measurement provides an excellent assessment of the quality of the growing season. Further, all covariates were standardized to indicate the percentage by which each was above or below 20 year averages. This facilitated model convergence and allowed us to compare the relative importance of each control on numbers of migrants.

Wolves (*Canis lupus*) were reintroduced to Yellowstone during 1995–1996, but we did not consider predation effects on out-of-the-park migrations by bison because wolves predominantly prey on elk (*Cervus elaphus*) [27] and, even in areas where wolf predation on bison is sometimes significant (e.g., Madison headwaters), we are unaware of any evidence for large-scale movements by bison in response to the presence of wolves [28]. We did not include predation effects by grizzly bears (*Ursus arctos*) since animals were predominantly in hibernation during the time of peak bison migrations.

We observed and/or handled all bison in compliance with the court-negotiated settlement for the Interagency Bison Management Plan [9], [10] and National Park Service research permit YELL-2008-SCI-5340, as well as guidelines recommended by the American Society of Mammalogists [29]. Field observation work included aerial counting of bison and is outlined in the Surveillance Plan for Yellowstone Bison (http://www.greateryellowstonescience.org/subproducts/121/7). Animal care and welfare procedures were approved by the National Park Service Veterinary Staff and are outlined in the Yellowstone Bison Management Capture and Handling Protocol (http://www.greateryellowstonesciece.prg/topics/biological/mammals/bison/projects/popdynamics).

### Model Development and Evaluation

We obtained posterior distributions for model parameters using Monte Carlo Markov chain methods in a hierarchal Bayesian framework. Our observed responses (Y_N,t_, Y_w,t_) were counts which were measured imperfectly, and the hierarchal framework allowed us to estimate posterior distributions of the unobserved, but true numbers of bison beyond park boundaries. We defined true annual maxima of bison beyond the northern park boundary as Z_N,t_ and western boundary as Z_W,t_.

#### Process Model

It is widely accepted that population size, snow, and forage availability affect movements of ungulates in temperate environments [30]–[32]. We anticipated that increasing bison population size and accumulated SWE would increase numbers migrating, and population size would interact with accumulated SWE such that larger incremental increases would occur with higher population size and snow measures. We hypothesized that increases in aboveground dried biomass may moderate the impetus for bison to move. Thus, our process equations included terms for population size, accumulated SWE, average aboveground dried biomass, and an interaction between population size and accumulated SWE.

We proposed alternative function forms of process equations representing competing ecological hypotheses of migration. A linear relationship was deemed infeasible because numbers migrating cannot exceed population size and numbers of bison exiting park boundaries far exceeded linear model predictions during 2000–2009. Only bison from the central herd have migrated outside the western park boundary, while bison from both the central and northern herds have migrated beyond the northern boundary (Figure 1). We began by using a logistic deterministic process equation portraying the probability that bison exit the north boundary
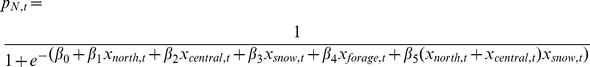
and west boundary
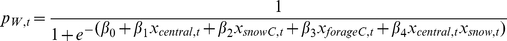



Bruggeman et al. [5] suggested that Yellowstone bison were partially migratory, with both migratory and resident components. We proposed the modified logistic process equation where *a* is a saturation parameter to represent this non-migrant component
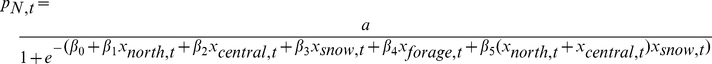



And
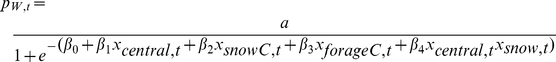



Bison may maintain a relatively stable winter density [15] and higher numbers may move beyond park boundaries under moderate covariate levels. Variations of the negative exponential functional form are often used in ecology to represent responses that initially increase and reach a plateau. We considered the negative exponential form portraying saturation as occurring at the population size 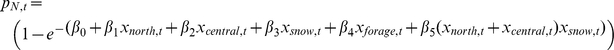
and
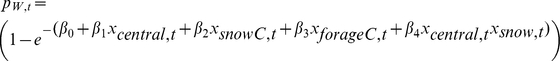



We also considered the modified negative exponential indicating saturation occurring at lower levels
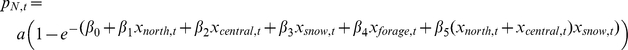
and
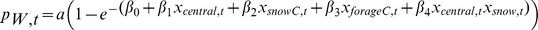



#### Process and Observation Model Stochasticity

Uncertainty in each process equation was included by treating Z_N,t_ and Z_W,t_ as binomial distributed random variables where
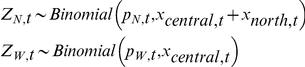



The binomial distribution is discrete and often used to model the number of successes in a sample of known size. Individual successes are not treated as independent, and we considered success as representing a bison that exited the park and failure as a bison that remained in the park [17]. We took the sample size of bison that may exit the north boundary as the sum of preceding summer counts of each herd (x_central,t_, x_north,t_) and west boundary as the preceding summer count of the central herd (x_central,t_) [12]. Uncertainty in observations was included by assuming observed responses (Y_N,t_, Y_W,t_) were also binomial distributed random variables such that
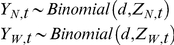
where d is a detection parameter. Here, we treated a success as an observation of a bison that exited the park.

#### Model Specification

We denoted **Y_N_** and **Y_W_** as vectors consisting of all annual observations, and **Z_N_** and **Z_W_** as vectors of process model predictions for all years. We also denoted **x_central_**, **x_north_**, **x_snow_**, **x_snowC_**, **x_forage_**, and **x_forageC_** as vectors of covariates. The prior distribution of *d* was provided by Hess [20] and we used uninformative prior distributions for other parameters. Likelihoods in the following model specification are easily identified as statements of states and observations conditional on parameters and covariates, and priors are statements of parameters conditional on distribution shape parameters. For convenience, we included the saturation parameter *a* in the following model specification, but this parameter was only present in the modified functional forms. The posterior distribution of migration beyond the northern boundary was specified as
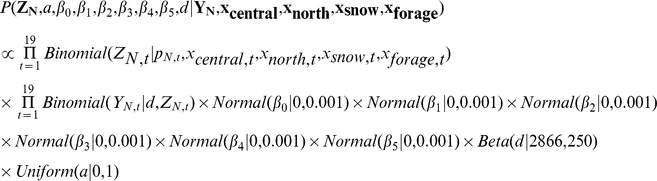
and beyond the western boundary as
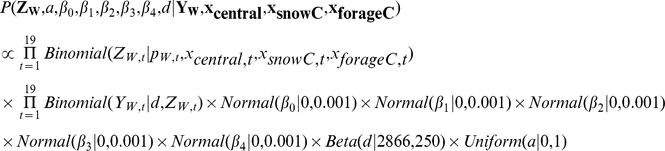



### Estimation and Model Selection

The Deviance Information Criterion (DIC) statistic [33] approximates the well-known Akaike Information Criterion (AIC) [34] statistic. Multi model-inference is inherently difficult and DIC has been criticized as being unreliable. DIC may bias support in higher parameterized models particularly when candidate models are hierarchal and priors are uninformative. Therefore, we instead made inferences using posterior distributions of model parameters and underlying process model predictions.

Monte Carlo Markov Chain procedures were implemented using the RJAGS package to call JAGS version 2.1.0 from R [35]. We ran each model for 50,000 iterations using three different Monte Carlo Markov chains. The first 10,000 iterations were excluded to allow for burn-in. We assessed convergence of chains using the Gelman and Heidelberg diagnostics using the gelman.diag and heidel.diag functions in R. We report posterior distributions of latent variables and parameters as 0.500 (median), and 0.025 and 0.975 quantiles (e.g. 95% credible intervals).

### Simulation Modeling of Migrations

We used our top models to simulate annual maxima of future migrations during 2010–2020 and considered alternate management scenarios. Annual accumulated SWE and aboveground dry biomass metrics were simulated using data collected during this study. We initialized central (1,800) and northern (2,000) herd sizes at known abundance during 2010. Annual growth in the absence of culling was simulated using a density-independent equation *_λ_*∼ Normal (1.07, 0.025) [36]. We estimated annual maxima of northern migrations as the median of posterior distributions. Since our model likely underestimated recent western migrations (see results), we estimated annual maxima of western migrations as the 90% quantile of posterior distributions. Migrations beyond the northern boundary generally occurred prior to western migrations. Therefore, we simulated northern migration and removed the appropriate number of bison according to each management scenario before simulating western migration and removing the appropriate number of western migrants. Out-of-park removals are conditional on several contingencies but, in general, allow bison migrating into Montana to be culled when the population exceeds 3,000 animals [9], [10]. Policies also stipulate increasing tolerance under smaller population sizes such that culls do not occur when there are fewer than 2,100 bison [9], [10]. We compared three removal scenarios during 2010–2020 and evaluated influences on numbers of bison migrating to the park boundary. Removals represented bison terminally exiting the population and can be viewed as a combination of transport of animals to quarantine facilities, harvest by hunters, and consignment to slaughter. Removals did not occur to a herd if members numbered <1,000 to satisfy collective preservation interests [13]. We considered 1) removing 50% of migrants; 2) removing up to 500 migrants; and 3) removing 100–150 bison from each herd annually. Approximately 40–60% of Yellowstone bison test positive for exposure to brucellosis and disease management policies stipulate culling of exposed migrants at park boundaries. Therefore, our 50% removal strategy coarsely portrayed the removal policy initially articulated in the Interagency Bison Management Plan. Our strategy that establishes an upper bound on removals represented selective removal of disease-exposed animals during large migrations and prevention of episodic removal of >20% of the population. Our fixed annual removal strategy represented limiting population growth.

## Results

The maximum number of bison counted at or beyond the northern park boundary summed with culls occurring prior to counting was highly variable during 1990–2009 (mean  = 326.5; sd  = 508.4; range: 0–1,979). Annual maxima occurred during February and March, and our measure of migration was generally fewer than 500 bison, other than during 1997 (899), 2006 (1,264), and 2008 (1,979). Peak numbers of bison migrating to the western boundary occurred during May and were smaller and more stable (mean  = 286.4; sd  = 163.9; range: 98–616). Northern and central herd counts were variable owing to episodic and large-scale removals, with numbers of bison in the central herd (1,399–3,531) exceeding numbers in the northern herd (455–2,070) before 2008. Annual forage estimates ranged from 216–666 g/m^2^ dried biomass and accumulated snow water equivalent estimates varied between 13 and 66 m.

The 0.975 quantile for Gelman potential scale reductions factors was <1.05 for all parameter estimates of logistic and modified logistic forms. MCMC chains for all parameters of these model forms passed Heidel tests of stationary distribution and for accuracy of the mean. The negative exponential and modified negative exponential forms of the underlying process equation violated convergence criteria and results are not reported.

**Figure 2:**
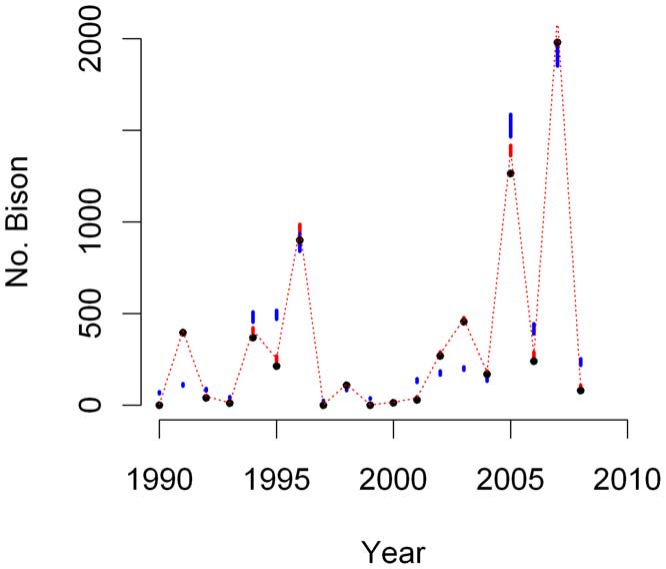
Modified logistic (red) predicted median (dotted lines) and 95% credible intervals (bars) of annual maxima of bison migrating beyond the northern boundary of Yellowstone National Park during 1990–2009. Observations (black circles) were precise (*d* = 0.92) resulting in narrow credible intervals of the vector of true states **Z_N_**. We plotted mean process model predictions (blue bars) as *p_N,t_*(**x_central_**+**x_north_**) to illustrate the relative contribution of process variance and observation error.

The logistic and modified logistic models performed similarly in evaluating numbers of bison migrating beyond the northern boundary of the park. The median of the saturation parameter (*a*) of the modified logistic model was 0.99 (Table 1) meaning the modified logistic model converged on the logistic model where we fixed the saturation parameter at one a priori. These results suggest that numbers migrating beyond the northern boundary saturate near total population size when central herd (e.g. >6,200) and northern herd (>2,800) sizes are much above 20-year averages. Also, 95% credible intervals of posterior distributions of parameters suggested high probabilities that each was either above or below zero meaning that covariate effects were in a specified direction (Table 1). There was a >95% probability that increases in central and northern herd sizes, and accumulated SWE increased numbers migrating beyond the northern park boundary. There was also a >95% probability that fewer bison migrated with increases in aboveground dried biomass. We did not estimate separate model parameters for process variance or observation error because we represented uncertainty using binomial distributions. However, a plot of process predictions of the modified logistic model compared to observed counts and predicted true states suggested excellent model performance (Figure 2).

**Table 1:** We estimated model parameters of competing hypotheses of annual maxima of bison migrating beyond the northern and western boundaries of Yellowstone National Park during 1990–2009.

NORTH			WEST		
	Logistic	Modified Logistic		Logistic	Modified Logisitc
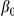	−2.79 (−2.83, −2.74)	−2.77 (−2.82, −2.71)	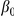	−1.99 (−2.02, −1.95)	−1.76 (−1.98, −1.01)
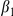	0.92 (0.75, 1.09)	0.92 (0.75, 1.10)	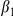	−0.62 (−0.75, −0.49)	−0.64 (−0.81, −0.50)
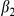	1.91 (1.83, 1.99)	1.92 (1.84, 2.00)	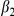	0.58 (0.50, 0.65)	0.60 (0.51, 0.71)
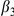	1.74 (1.67, 1.82)	1.74 (1.67, 1.82)	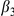	−0.60 (−0.72, −0.46)	−0.62 (−0.77, −0.48)
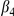	−1.05 (−1.22, −0.88)	−1.05 (−1.22, −0.88)	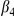	0.39 (0.18, 0.61)	0.38 (0.14, 0.61)
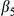	−0.85 (−1.17, −0.53)	−0.85 (−1.17, −0.53)	*a*		0.82 (0.45, 0.99)
*a*		0.99 (0.95, 1.00)	*d*	0.92 (0.91, 0.93)	0.92 (0.91, 0.93)
*d*	0.92 (0.91, 0.93)	0.92 (0.91, 0.93)			

Point estimates represent medians and ranges are 95% credible intervals of posterior distributions. Abbreviations for models of north migration are intercept (β_0_), central herd size (β_1_), northern herd size (β_2_), accumulated SWE (β_3_), aboveground dried biomass (β_4_), interaction between the sum of herd sizes and accumulated SWE (β_5_), saturation (*a*), and count detection (*d).* Abbreviations for models of west migration are intercept (β_0_), central herd size (β_1_), accumulated SWE (β_2_), aboveground dried biomass (β_3_), interaction between the central herd size and accumulated SWE (β_4_), saturation (*a*), and count detection (*d)*. The negative exponential and modified negative exponential models violated convergence criteria and results are not reported.

**Figure 3:**
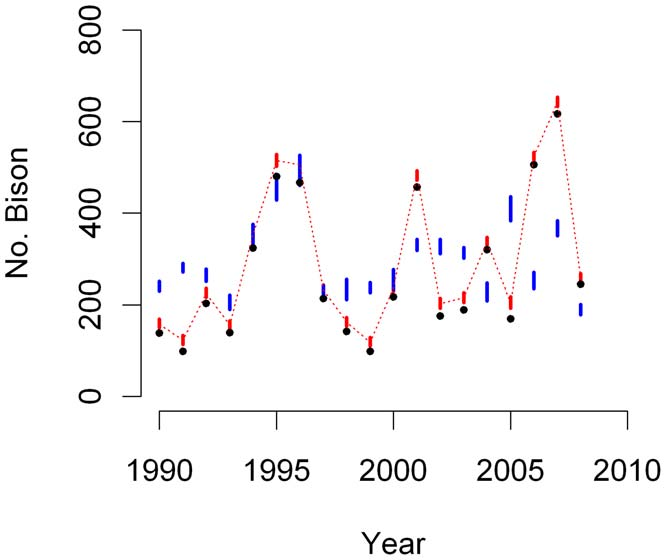
Modified logistic (red) predicted median (dotted lines) and 95% credible intervals (bars) of annual maxima of bison migrating beyond the western boundary of Yellowstone National Park during 1990–2009. Observations (black circles) were precise (*d* = 0.92) resulting in narrow credible intervals of the vector of true states **Z_W_**. We plotted mean process model predictions (blue bars) as *p_W,t_* **x_central_** to illustrate the relative contribution of process variance and observation error.

Contrary to the north response, the median of the saturation parameter of the modified logistic form was 0.82 providing support that not all central herd animals exit the western boundary when central herd size (>6,200) is much above the 20-year average. We found a >95% probability of greater numbers moving beyond the western boundary with increases in central herd size, increases in accumulated SWE, and decreases in aboveground dried biomass (Table 1). We plotted process predictions of the modified logistic model compared to observed counts and predicted true states, and model performance declined beginning around 2001 suggesting that an important control on recent western migration was excluded (Figure 3).

Simulation modeling of future migrations indicated that large and episodic migrations of bison beyond the northern and western boundaries of Yellowstone would occur during the next decade regardless of the management scenario. If half of all migrants are culled and herds are maintained above 1,000 members, we predict ≥250 bison will exit the northern boundary during 7.79 (SD  = 1.27), ≥500 bison during 4.37 (1.02), and ≥1,000 bison during 1.24 (0.64) of the next ten years. We also predict ≥250 migrants exiting the western park boundary during 1.13 (0.73) of the next ten years. Assuming removals are targeted towards bison exposed to brucellosis, our models indicate that several hundred susceptible and/or vaccinated migrants may need to be tolerated outside the park during certain winters to support current brucellosis management strategies. Further, a strategy of limiting population growth through consistent annual reductions of 100–150 bison from each herd resulted in increased regularity and magnitude of out-of-park migrations. Beyond the northern boundary, we predict ≥250 animals during 9.95 (0.22), ≥500 animals during 9.62 (0.71), and ≥1,000 animals during 6.84 (2.30) of the next ten years. We also predict ≥250 animals outside the western boundary during 5.46 (1.80) of the next ten years.

Removing up to 500 migrants comparatively reduced the frequency of small and moderate migrations beyond the northern boundary with predicted migrations of ≥250 animals during 5.96 (0.97) and ≥500 animals during 3.43 (1.01) of the next ten years. Predicted large migrations of ≥1,000 animals occurred during 1.33 (0.76) of the next ten years. This strategy may complicate brucellosis management by removing susceptible individuals when there are insufficient numbers of migrants to selectively remove bison testing positive for brucellosis exposure. However, setting an upper bound on removals prevents the episodic removal of >20% of the population and reduces the frequency and magnitude of future migrations into Montana.

## Discussion

Few opportunities exist to evaluate the unimpeded migration of large ungulates across expansive and heterogeneous landscapes unaltered by anthropogenic disturbance [37]. Seasonal migrations of bison in Yellowstone have been reestablished after near extirpation during the early 20^th^ century and we cannot be sure that current movement patterns reflect historic spatial dynamics. We demonstrated that migration differed at the scale of herds, but were able to predict migrations by both herds using a single unifying model that provided insights into the underlying processes. Nonlinear responses of migratory ungulates to snow [38] and vegetation [39] are receiving increased attention [40] and, to our knowledge, this is the first evidence that the relationship between bison migration, climate, and density is logistic in form.

Recent movements by bison beyond the north boundary challenge the idea that the area occupied by bison expands with population size to maintain a relatively stable winter density [4], [15]. If that were the case, we would expect stronger support for the negative exponential model form which represents increases in numbers exiting the park beginning at lower herd sizes. Instead, we found high probability that fewer than 10 percent of the population exited the park under moderate levels of herd size (1,000–2,000), accumulated SWE (<60%), and aboveground dried biomass (>100%), above which numbers exiting rapidly increased (Table 2). We provide continued evidence of snow and herd size acting as controls on movements, [4]–[7], [15] and show that forage production affects migrations.

**Table 2:** Predicted annual maxima of bison migrating beyond the northern and western boundaries of Yellowstone National Park generated using a modified logistic process equation that incorporates the effects of central herd and northern herd size, accumulated SWE, aboveground dried biomass, and an interaction between herd size and accumulated SWE.

NORTH BOUNDARY										
Central	Northern	Snow	60%	60%	100%	100%	100%	130%	130%	130%
		Forage	100%	60%	130%	100%	60%	130%	100%	60%
1,000	1,000	—	135	185	210	275	360	390	500	635
1,500	1,000	—	175	240	250	335	440	460	580	740
2,000	1,000	—	215	285	305	400	525	550	700	890
1,000	1,500	—	255	340	320	415	535	510	630	785
1,500	1,500	—	330	445	410	520	675	605	765	960
2,000	1,500	—	400	530	485	625	810	725	915	1,150
1,000	2,000	—	470	600	510	645	810	690	860	1,040
1,500	2,000	—	620	790	650	830	1,040	870	1,070	1,300
2,000	2,000	—	740	950	785	1,000	1,250	1,040	1,290	1,560

WEST BOUNDARY										
Central	Northern	Snow	60%	60%	100%	100%	100%	130%	130%	130%
		Forage	100%	60%	130%	100%	60%	130%	100%	60%
1,000	—	—	150	170	140	160	185	155	180	205
1,500	—	—	235	270	255	295	335	315	360	415
2,000	—	—	260	300	260	300	345	295	340	390

Table values indicate approximate maxima abundances with 95% probability, e.g. the probability that there will be no more than the listed number of bison outside of the park is 0.95 given central and northern herd sizes, and accumulated SWE (snow) and aboveground dry biomass (forage) as percentages of 20-year averages.

We evaluated a separate response for migration beyond the western park boundary where the logistic model did not perform as well. Numbers of bison remaining in high elevation summering valleys through mid-winter stabilized as the central herd increased in size – suggesting partially migratory tendencies [5]. The timing of migrations may be delayed as peak numbers of bison outside the western boundary occur during April and May. Migration during the growing season is driven by selection for high quality forage in a variety of ungulates, particularly when nutritional requirements associated with reproduction are peaking and animals are likely seeking out regions with emerging vegetation to provide high quality milk for offspring [41]. Central herd bison may exploit new grass growth outside the park while the high-elevation summer ranges are still covered with snow [42].

The process variance term in our models represents all controls on underlying movement processes that were excluded. While it is impossible to retrospectively determine effects, bison movements were undoubtedly influenced by more than a century of management actions and human-induced alterations to the environment. Management of bison along the western park boundary during 2000–2005 predominantly involved aggressive hazing of animals back into the park as opposed to the northern boundary where thousands of migrants were culled or held in containment pens. Movements of central herd animals to the northern range increased during this time [12], and perhaps bison that were repeatedly hazed sought alternate routes to lower elevation wintering areas. More recently, aggressive hazing of bison outside the western boundary has been delayed until late April and observed numbers of bison outside the western boundary increased. The Bayesian framework handles such behavioral plasticity by using an iterative process of understanding where past observations are incorporated with newly collected data, and with time we may identify such relationships.

If migration by bison into Montana is restricted by forcing bison to remain within the park, or shortened by hazing animals back into the park before spring forage conditions are suitable, then bison numbers would ultimately be regulated by food availability within Yellowstone and the bison population would reach high densities before substantial winterkill occurs [43]. These high densities of bison could cause significant deterioration to other park resources (e.g. vegetation, soils, and other ungulates) and processes as the bison population overshoots their food capacity within the park. Alternatively, migrating bison have been culled. Recurrent, large-scale culls of bison occurred with >1,000 bison culled from the population during winters 1997 (21%) and 2006 (32%), and >1,700 bison (37%) culled during winter 2008.

Plumb et al. [14] recommended maintaining the bison population between 2,500–4,500 to satisfy collective interests concerning the park's forage base, bison movement ecology, retention of genetic diversity, brucellosis risk management, and prevailing social conditions. We showed that migrations are predictable, but the magnitudes of migrations are highly influenced by uncontrollable variables such as snow pack severity and plant production. When accumulated SWE is 150% of the 20-year average, aboveground dry biomass is 50% of the 20-year average, and there are 2,500 bison (1,250 central and 1,250 northern) in the population, we predict a 95% probability (e.g. chance) of ≤1,135 animals migrating beyond the northern and ≤300 animals migrating beyond the western park boundaries. Density exacerbates movements and under similar severe climate conditions and 4,500 (2,500 central and 2,000 northern) bison in the population, we predict a 95% chance of ≥1,820 animals exiting the north boundary. Dramatically fewer bison migrate under more moderate climate conditions even when there are 4,500 bison due to the logistic form of the migration response (Table 2). Thus, potential migrations range from few individuals to thousands of bison in any year when the population is within the recommended range of 2,500–4,500 animals.

Yellowstone's restored bison herds have established migratory patterns that lead them to low elevation areas out of the park where they come into conflict with society. Our simulation results suggest scenarios that remove 50% of migrants similar to management policies outlined in the Interagency Bison Management Plan will not prevent future large-scale, recurrent migrations and numbers exiting park boundaries will be much greater than predictions underlying those policies. Thus, limiting bison numbers and allowing increased numbers of bison beyond park boundaries during severe climate conditions may be the only means of avoiding episodic, large-scale reductions to the Yellowstone bison population in the foreseeable future. Limiting bison abundance to lower numbers will likely reduce (but not eliminate) the frequency of large-scale migrations into Montana, but could also hamper the conservation of this unique population of wild, free-ranging bison by adversely affecting the population's resiliency to respond to environmental challenges, genetic diversity, and the ecological role of bison in the ecosystem through the creation of landscape heterozygosity, nutrient redistribution, competition with other ungulates, prey for carnivores, habitat creation for grassland birds and other species, provision of carcasses for scavengers, stimulation of primary production, and opened access to vegetation through snow cover [1], [13], [14].
